# Genome scan linkage analysis comparing microsatellites and single-nucleotide polymorphisms markers for two measures of alcoholism in chromosomes 1, 4, and 7

**DOI:** 10.1186/1471-2156-6-S1-S4

**Published:** 2005-12-30

**Authors:** Guanjie Chen, Adebowale Adeyemo, Jie Zhou, Ao Yuan, Yuanxiu Chen, Charles Rotimi

**Affiliations:** 1 National Human Genome Center, College of Medicine, Howard University, Washington, DC, USA

## Abstract

**Background:**

We analyzed 143 pedigrees (364 nuclear families) in the Collaborative Study on the Genetics of Alcoholism (COGA) data provided to the participants in the Genetic Analysis Workshop 14 (GAW14) with the goal of comparing results obtained from genome linkage analysis using microsatellite and with results obtained using SNP markers for two measures of alcoholism (maximum number of drinks -MAXDRINK and an electrophysiological measure from EEG -TTTH1). First, we constructed haplotype blocks by using the entire set of single-nucleotide polymorphisms (SNP) in chromosomes 1, 4, and 7. These chromosomes have shown linkage signals for MAXDRINK or EEG-TTTH1 in previous reports. Second, we randomly selected one, two, three, four, and five SNPs from each block (referred to as Rep1 – Rep5, respectively) to conduct linkage analysis using variance component approach. Finally, results of all SNP analyses were compared with those obtained using microsatellite markers.

**Results:**

The LOD scores obtained from SNPs were slightly higher but the curves were not radically different from those obtained from microsatellite analyses. The peaks of linkage regions from SNP sets were slightly shifted to the left when compared to those from microsatellite markers. The reduced sets of SNPs provide signals in the same linkage regions but with a smaller LOD score suggesting a significant impact of the decrease in information content on linkage results. The widths of 1 LOD support interval of linkage regions from SNP sets were smaller when compared to those of microsatellite markers. However, two linkage regions obtained from the microsatellite linkage analysis on chromosome 7 for LOG of TTTH1 were not detected in the SNP based analyses.

**Conclusion:**

The linkage results from SNPs showed narrower linkage regions and slightly higher LOD scores when compared to those of microsatellite markers. The different builds of the genetic maps used in microsatellite and SNPs markers or/and errors in genotyping may account for the microsatellite linkage signals on chromosome 7 that were not identified using SNPs. Also, unresolved map issues between SNPs and microsatellite markers may be partly responsible for the shifted linkage peaks when comparing the two types of markers.

## Background

The identification of chromosomal segments showing association or linkage is only the first step toward discovery of genetic factors underlying susceptibility to disease. The typical genome-wide linkage analysis based on microsatellites with an average density of 10 cM results in large genomic regions for fine-mapping. In this regard, there is considerable interest in developing maps based on genomic markers that will lead to higher resolution linkage results with the hope of reducing future cost and time to conduct fine-mapping. With the availability of several million new SNPs in the public database and new technologies for large-scale, high throughput SNP genotyping at affordable costs, there is growing interests in using SNPs to create high resolution linkage maps. In this paper we evaluate strategies to systematically compare genome-wide linkage results from microsatellite and SNPs using different density maps.

## Methods

### Materials

The dataset for the Collaborative Study on the Genetics of Alcoholism (COGA) was provided as problem 1 for GAW14. The dataset included 1,350 individuals in 143 pedigrees, 318 microsatellite genotypes for a 10 cM genome map, 4,763 SNP loci from Illumina, 11,555 SNP loci from Affymetrix and phenotypic information. We used MAXDRINK and TTTH1 as phenotypes and the panel of 4,763 Illumina SNPs. MAXDRINK is defined as maximum number of drinks in a 24-hour period [1] and TTTH1 is defined as the Visual Oddball Experiment and the Eyes Closed Resting EEG dataset for frontal left side channel. The extracted measures correspond to the 'late' time window, which is set at 300 to 700 ms following stimulus presentation (bounding the visual P3 event), and the theta band power (3 to 7 Hz) [2]. These phenotypes were log transformed for all analyses. Three chromosomes (1, 4, and 7) which show linkage signals for MAXDRINK or TTTH1 phenotypes in previous reports [1,2] were selected for our analyses.

### Statistical analysis

For each chromosome, we constructed haplotypes using GENEHUNTER2 (GH2) [3]. Linkage equilibrium among markers is assumed in GH2. As discussed by Shaid D.J. et al. [4], if closely spaced markers are useful for haplotype fine mapping, it is reasonable to assume that that the markers themselves are in linkage disequilibrium (LD), because the implicit basis of fine mapping by haplotypes is LD. Haplotype blocks were generated using the statistical framework method [5], in which the inference on the optimal haplotype block partitioning is formulated as the problem of statistical model selection based on the likelihood of the observed data to define regions with a very small proportion of comparisons among informative SNP pairs showing strong evidence of historical recombination. We selected SNPs, at random, from each block to test for the minimum number of SNPs required to achieve the same results as using all the SNPs in a block. Rep1 represents the process of randomly selecting one SNP from a block and Rep2 for randomly selecting 2 SNPs from a block; this process was repeated until we selected the maximum of 5 SNP (Rep5) from each block. We stoped at five because the minimum observed number of SNPs in observed blocks was 5. We also conducted linkage analysis using all available SNPs. A variance components approach as implemented in SOLAR was used for all analyses [6]. The linkage results using microsatellites markers were then compared to those from constructed haplotype blocks and for reduced number of SNPs from each block (Rep1 through Rep5) and entire set of SNPs. The range of positional candidate regions was defined by a logarithm of odds (LOD) score of ≥ 1.0.

## Results

**Figure 1:**
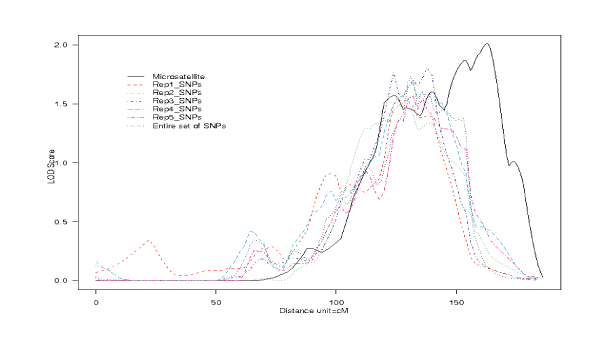
**Linkage plot for the log transformed TTTH1 phenotype in chromosome 7. **LOD scores for microsatellite (*solid line*) and SNPs (*dashed lines*). The different colored dashed lines represent the results of the linkage analyses based on randomly selected one, two, three, four, and five SNPs from each haplotype blocks and using the entire set of SNPs.

**Figure 2:**
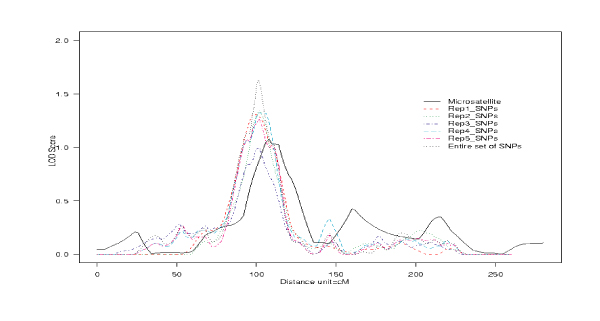
**Linkage plot for the log transformed MAXDRINK phenotype in chromosome 1. **LOD scores for microsatellite (*solid line*) and SNPs (*dashed lines*). The different colored dashed lines represent the results of the linkage analyses based on randomly selected one, two, three, four, and five SNPs from each haplotype blocks and using the entire set of SNPs.

The residual kurtosis of LOG transformed MAXDRINK and TTTH1 are -0.18 and 0.57, respectively allowing the assumption of normality in our analyses. The distribution of haplotype blocks for chromosome 1, 4, and 7 are displayed in Table 1. Although the LOD scores from the linkage analyses based on SNPs, as compared with microsatellites, were consistently larger (*p *< 0.01), the location of the signals were for the most part similar (Figures 1 and 2). Interestingly, two linkage regions on chromosome 7 (154 cM and 163 cM) were not detected in the SNP analyses for the TTTH1 phenotype (Table 2 and Figure 1). The SNP density and associated information content around the chromosome 7 linkage peaks using STRP are displayed in table 3. No significant linkage signals were observed for chromosome 4. Overall, the largest LOD score of 1.66 was observed on chromosome 1 for the analyses based on entire set of SNPs using the log of MAXDRINK as the phenotype (Table 2). Table 4 shows the widths and boundaries of linkage regions in chromosome 7 for LOG TTTH1 and chromosome 1 for LOG MAXDRINK. Width of linkage regions for LOG TTTH1 was 58 cM from microsatellite markers, compared with 24 cM, 40 cM, 34 cM, 3 8 cM, 30 cM, and 33 cM, respectively, from Rep1 to Rep5 and the entire set of SNPs.

## Discussion

In all, the patterns of linkage results from microsatellites were similar to those obtained from SNPs analyses for chromosome 1, 4, and 7. It was however notable that the SNP analyses did not detect two linkage regions on chromosome 7 (LOD = 1.87 and 2.01; Table 2). As displayed in Figures 1 and 2, the LOD score peaks generated from SNPs were slightly shifted to the left when compared to that from microsatellite markers. A potential reason for this observation may be the different builds of the genetic maps used for the microsatellite markers and SNPs, and/or errors in genotyping [7]. Kruglyak [8] observed an increase in LOD scores for a proportionate increase in the information content of linkage map as derived from a denser SNP map. In our results, reducing the number of SNPs in each block to 1, 2, 3, 4, and 5 SNPs did not significantly change the shape of linkage signals albeit a small drop in peak height. Since expected LOD scores correlate with information content, from table 3, there is only a small reduction in information contents for Rep1 and others are the same. It has been estimated that 1.7–2.5 SNP markers provide equivalent information as one microsatellite marker [8,9] and that a 10 K SNP array provides at least equal power to detect linkage compared with a search based upon a 5 Mb microsatellite screen [10]. In our results, 2.5 SNP markers provide equal information content as one microsatellite marker. These observations support the idea that the use of high dense SNP maps for performing linkage analysis should result in more precisely defined loci at substantially reduced cost.

## Conclusion

The linkage results from SNP maps can result in narrower linkage regions with higher LOD scores when compared with microsatellite marker maps. The linkage results from reduced sets of SNPs provided signals in the same linkage regions but with a smaller LOD scores, suggesting that loss of information content influenced expected LOD scores. The different builds of the genetic maps used in microsatellite markers and SNPs or/and errors in genotyping may have led to the significant linkage region observed on chromosome 7 in the microsatellite scan that was not detected in the genome scan based on SNPs, and for peaks from SNPs being slightly shifted to the left of the microsatellite peaks.

## Abbreviations

COGA: Collaborative Studies of the Genetics of Alcoholism (COGA)

GAW: Genetic Analysis Workshop 14

SNP: Single-nucleotide polymorphisms

STRP: Short tandem repeat polymorphism

## Figures and Tables

**Table 1:** Haplotype block distributions for chromosomes 1, 4, and 7

		No. SNPs			Length of block (cM)		
			
Chromosome	No. blocks	Minimum	Maximum	Average	Minimum	Maximum	Average
1	55	5.0	12	6.93	0.30	18.2	4.72
4	37	5.0	16	7.27	0.70	18.4	5.49
7	35	5.0	17	7.60	0.90	26.0	5.28
Total	127	5.0	17	7.21	0.30	26.0	5.10

**Table 2:** Significant linkage results

		LOD score (location)						
		
			Replicate^a^					
				
Chr	Phenotype	Microsatellites	1	2	3	4	5	Entire set of SNPs
7	LOG of TTTH1	**1.57^b ^(124 cM)**	**1.49 (124 cM)**	**1.44 (124 cM)**	**1.77 (124 cM)**	**1.50 (124 cM)**	**1.24 (124 cM)**	**1.27 (124 cM)**
7	LOG of TTTH1	**1.47 (131 cM)**	**1.50 (131 cM)**	**1.55 (131 cM)**	**1.70 (131 cM)**	**1.67 (131 cM)**	**1.56 (131 cM)**	**1.73 (131 cM)**
7	LOG of TTTH1	**1.43 (136 cM)**	**1.38 (136 cM)**	**1.30 (136 cM)**	**1.72 (136 cM)**	**1.58 (136 cM)**	**1.57 (138 cM)**	**1.61 (136 cM)**
7	LOG of TTTH1	**1.87 (154 cM)**	0.35 (154 cM)	0.57 (154 cM)	0.58 (154 cM)	0.91 (154 cM)	**1.12 (154 cM)**	**1.25 (154 cM)**
7	LOG of TTTH1	**2.01 (163 cM)**	0.10 (163 cM)	0.29 (163 cM)	0.13 (163 cM)	0.43 (163 cM)	0.42 (163 cM)	0.11 (163 cM)
1	LOG of MAXDRINK	0.91 (103 cM)	**1.26 (103 cM)**	**1.32 (103 cM)**	**1.00 (103 cM)**	**1.33 (103 cM)**	**1.26 (103 cM)**	**1.66 (103 cM)**

^a^Replicates: 1, one SNP randomly selected from each block; 2, two SNPs randomly selected from each block; 3, three SNPs randomly selected from each block; 4, four SNPs randomly selected from each block; 5, five SNPs randomly selected from each block.

^b^Bold text indicates LOD score > 1.0.

**Table 3:** SNP density and information content around the chromosome 7 linkage peak (114–172 cM) for STRP scan

Classification of genome scans	No. STRP or SNPs	Information content mean (SD)
Microsatellites	10	0.87 (0.04)
Rep1^a^	12	0.65 (0.06)
Rep2^a^	25	0.86 (0.06)
Rep3^a^	38	0.90 (0.04)
Rep4^a^	51	0.91 (0.06)
Rep5^a^	61	0.92 (0.04)
Entire set of SNPs	96	0.93 (0.03)

^a^Replicates: 1, one SNP randomly selected from each block; 2, two SNPs randomly selected from each block; 3, three SNPs randomly selected from each block; 4; four SNPs randomly selected from each block; 5, five SNPs randomly selected from each block.

**Table 4:** Widths of linkage regions by chromosome

	Width (boundaries)	
	
Classification of genome scan	7/LOG of TTTH1	LOG of MAXDRINK
Microsatellites	58 cM (114–172 cM)	8 cM (106–114 cM)
Replicate 1^a^	24 cM (120–144 cM)	8 cM (92–110 cM)
Replicate 2^a^	40 cM (108–148 cM)	12 cM (96–108 cM)
Replicate 3^a^	34 cM (112–146 cM)	1 cM (101–102 cM)
Replicate 4^a^	38 cM (114–152 cM)	8 cM (92–110 cM)
Replicate 5^a^	30 cM (124–154 cM)	14 cM (93–107 cM)
Entire set of SNPs	33 cM (121–154 cM)	8 cM (93–111 cM)

^a^Replicates: 1, one SNP randomly selected from each block; 2, two SNPs randomly selected from each block; 3, three SNPs randomly selected from each block; 4, four SNPs randomly selected from each block; 5, five SNPs randomly selected from each block.
